# Optically trapped room temperature polariton condensate in an organic semiconductor

**DOI:** 10.1038/s41467-022-34440-0

**Published:** 2022-11-23

**Authors:** Mengjie Wei, Wouter Verstraelen, Konstantinos Orfanakis, Arvydas Ruseckas, Timothy C. H. Liew, Ifor D. W. Samuel, Graham A. Turnbull, Hamid Ohadi

**Affiliations:** 1 Organic Semiconductor Centre, School of Physics & Astronomy, SUPA, University of St Andrews, St Andrews, KY16 9SS UK; 2 Division of Physics and Applied Physics, School of Physical and Mathematical Sciences, Nanyang Technological University, Singapore, Singapore

**Keywords:** Polaritons, Solid-state lasers

## Abstract

The strong nonlinearities of exciton-polariton condensates in lattices make them suitable candidates for neuromorphic computing and physical simulations of complex problems. So far, all room temperature polariton condensate lattices have been achieved by nanoimprinting microcavities, which by nature lacks the crucial tunability required for realistic reconfigurable simulators. Here, we report the observation of a quantised oscillating nonlinear quantum fluid in 1D and 2D potentials in an organic microcavity at room temperature, achieved by an on-the-fly fully tuneable optical approach. Remarkably, the condensate is delocalised from the excitation region by macroscopic distances, leading both to longer coherence and a threshold one order of magnitude lower than that with a conventional Gaussian excitation profile. We observe different mode selection behaviour compared to inorganic materials, which highlights the anomalous scaling of blueshift with pump intensity and the presence of sizeable energy-relaxation mechanisms. Our work is a major step towards a fully tuneable polariton simulator at room temperature.

## Abstract (web summary)

The authors demonstrate on-the-fly reconfigurable optical trapping of organic polariton condensates which are delocalised over a macroscopic distance from the excitation region, holding great potential for future work on polaritonic lattice physics.

## Introduction

Exciton–polaritons are formed due to strong coupling between excitons and cavity photons^1^. They are part-light, part-matter bosonic quasi-particles that inherit properties from their constituents: the photonic constituent results in small effective mass and high-speed propagation over macroscopic distances, while the excitonic fraction brings the nonlinearity^2,3^. These characteristics allow for higher temperature condensation (compared to ultracold atomic gases), which has revealed properties that resemble atomic Bose–Einstein condensates and superfluids^4–6^. Archetypal polariton-based devices rely on strong nonlinearities^7^ and ultrafast velocities of polaritons^8^, as well as engineering the potential landscape, which is crucial to manipulating, tailoring, and directing the polariton flow^9^. By engineering the potential, various applications of polariton condensates have been realised, such as an analogue simulator for classical *XY* Hamiltonians^10^, topological Chern insulators^11^, neural networks^12^, and electrically driven spin-switches^7^.

As a hybridisation of exciton and photon, polaritons can be trapped via either of the two components. To trap polaritons via their photonic part, lateral confinement is normally exploited, including applying a metal mask to a grown microcavity^13^, etched micropillar cavities^14^, and photonic crystal cavities^15^. Additionally, the excitonic component allows for controllable post-fabrication potentials, including the application of surface acoustic waves^16^ and local strain^5^, and sculpting an exciton reservoir by spatially modulating the optical pump^17^. To precisely control the polariton energy landscape, highly advanced nanotechnology is required in most cases. Among all these confinement methods, all-optical trapping of polaritons via shaping the non-resonant pump beam profile has been found to be a very elegant and flexible way to manipulate and steer polaritons^17–25^. Although polaritons and their condensates have been observed in various material systems, ranging from inorganic II–VI^4^, III–V semiconductors^26^, perovskites^27^, layered materials^28^, and organic semiconductors^29^, the majority of implementations of controlled potentials for polariton condensates were carried out in GaAs cavities, which need to operate at liquid helium temperature.

In this work, we utilised an organic semiconductor possessing tightly bound Frenkel excitons with high exciton binding energy and large oscillator strength, sustaining polaritons and their condensate at room temperature^30^. Optical trapping of polaritons was implemented using a spatial light modulator (SLM) to shape the non-resonant pump beam into 1D and 2D potential landscapes^17^. By optically imprinting 1D parabolic-like potential wells using two pump spots with tuneable separation, quantised states were observed clearly at room temperature. Square-shaped 2D confinement of polaritons enabled the formation of a trapped condensate that was partially separated from the exciton reservoir over macroscopic distances, resulting in reduced threshold and energy linewidth of condensation. By further increasing the width of the 2D potential, polaritons can be trapped in higher excited states. Our system is a key demonstration of a room-temperature quantum fluid, where the flow and the state can be controlled on demand.

## Results

### 1D optical trapping of organic polaritons

**Fig. 1:**
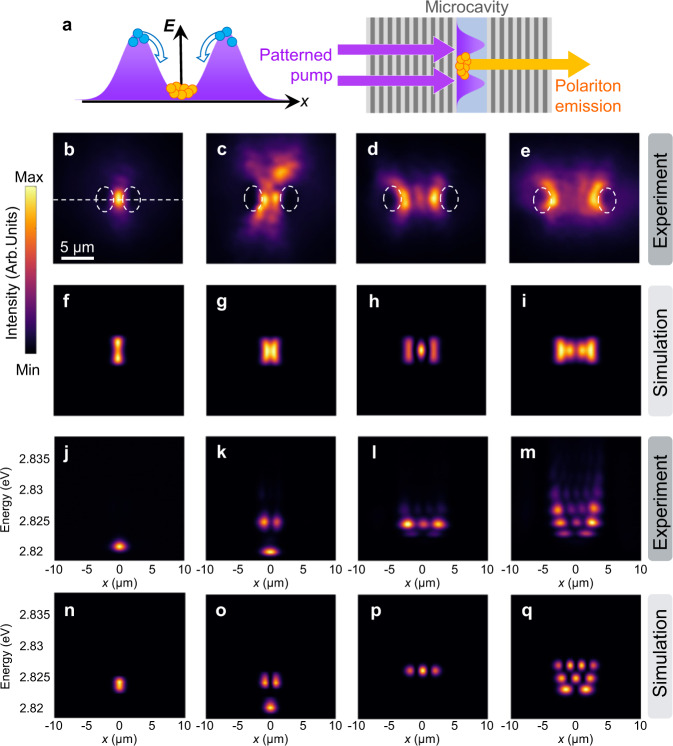
Schematic structure and characterisation of one-dimensional trapping. **a** Experimental scheme with the patterned pump incident on the substrate of a planar microcavity and the emission collected from the cavity surface. The left image shows two neighbouring Gaussian beams forming a 1D potential. The arrows denote the dominant flows of polaritons toward the bottom of the potential. **b–e** Experimental and **f–i** theoretical real-space images of condensate emission for two 6.6 μm × 4.5 μm pump spots separated by 4.1 μm (**b**, **f**); 5.6 μm (**c**, **g**); 8.8 μm (**d**, **h**); and 10 μm (**e**, **i**). White dashed ellipses indicate the pump spots. **j–m** Corresponding experimental and **n–q** theoretical real-space spectra along the centre of real-space images indicated by the dashed line in (**b**).

The studied sample is a *λ*/2 microcavity, consisting of two distributed Bragg reflectors (DBR) filled by a thin layer of the conjugated polymer, poly(9,9-dioctylfluorene) (PFO) (see Fig. 1a, Fig. S1 and the “Methods” section for details). We have previously demonstrated strong coupling and condensation in the same sample^30^. An SLM is used to project two neighbouring Gaussian pump spots (diameters, 6.6 μm × 4.5 μm) with similar intensity profiles onto the substrate side of the cavity at normal incidence, while the emission from the cavity is collected from the sample surface. The non-resonant pump creates a localised exciton reservoir and population of polaritons around the pump region, inducing a parabolic-like optical potential (Fig. 1a). At a very low pump fluence, only incoherent emission is observed at the pump location (Fig. S2). The real space image of emission from the PFO cavity pumped above the threshold by two spots separated by 4.1 μm shows that the polariton condensate is apparently confined in the potential well formed by the two pump spots (Fig. 1b). By spectrally resolving the profile along the centre of the trapped spatial mode, a single polariton condensate is observed with energy at 2.82 eV (Fig. 1j). By increasing the separation between the two pump spots, an increasing number of spatial modes can be resolved (Fig. 1c–e). Correspondingly, quantised polariton energy levels are seen in the spatially resolved spectra (Fig. 1k–m, see Fig. S3 for more separations). For larger separations, we observe that polaritons condense into higher excited states above the threshold. Introducing the 1D potential trap leads to the redistribution of polaritons in both energy and space. To better understand the trapping mechanism and the formation of the higher excited states, we have modelled our polariton system.

The behaviour of trapped polaritons can be theoretically modelled with a macroscopic wavefunction $\psi \left({{{{{\bf{x}}}}}}\right)$, in accordance with the standard mean-field theory for Bose–Einstein condensation. Its coherent time evolution is governed by a Gross–Pitaevskii equation (GPE)^31^

1 $$i{{\hslash }}{\left.\frac{{\rm {d}}\psi }{{{\rm {d}}t}}\right|}_{{{\rm {coh}}}}={\hat{H}}_{\psi,{{{{{\bf{x}}}}}}} \psi,$$

where

2 $${\hat{H}}_{\psi,{{{{{\bf{x}}}}}}}=-\frac{{{\hslash }}^{2}{\nabla }^{2}}{2m}+V\left({{{{{\bf{x}}}}}}\right)+g{\left|\psi \left({{{{{\bf{x}}}}}}\right)\right|}^{2}.$$

Here, *V* is the pump-induced potential, proportional to the spatial pump profile *P*(**x**). Physically, such a term can be anticipated from the saturation of the exciton oscillator strength resulting in the reduction of strong coupling. *g* is the nonlinear repulsive interaction between the particles (analogous to the Kerr effect for the optical case). We will typically neglect this term (*g* = 0) as it is known that this effect is weak for organic polaritons, although we will briefly reconsider this point below. This property of low nonlinearity marks a crucial difference with typical inorganic exciton-polaritons: the exciton component of the polaritons considered here is of Frenkel rather than Wannier–Mott nature^32^.

The simulations of the GPE show a good agreement with the experiment (Fig. 1f–i and n–q). For the simulations, we diagonalised Eq. (2) on a grid (see the “Methods” section) and assigned weights to the different modes matching experimental intensities. The potential profile induced by each pump spot was extracted to be a Gaussian with a height of 20 meV in all cases. We note that this potential is not due to the repulsive interaction between excitons, but due to the change in the polariton energy because of bleaching. The bleaching reduces the oscillator strength, which in return reduces the Rabi splitting and shifts the lower polariton up and creates an effective “blueshift” potential^32^. A varying pump beam width was employed for simulations, namely 4 μm for separations of 4.1 and 5 μm, and 4.5 μm for the remaining separations, to correspond to minor variations of pump width in the experiment (Fig. S2).

**Fig. 2:**
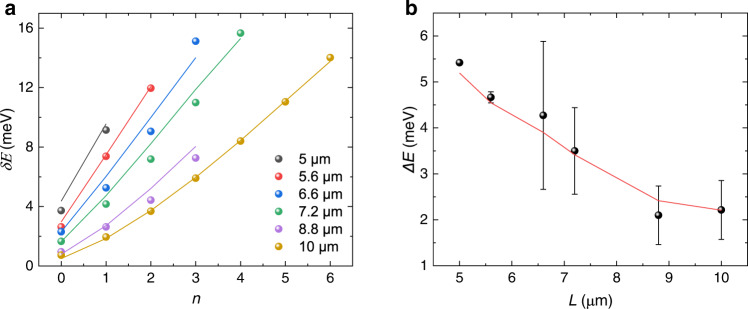
Dependence of quantised states on pump separations. **a** Extracted relative mode energies *δE* versus quantum number *n* for multiple separations between two pump spots. Solid circles and lines represent experimental and theoretical results, respectively. **b** Average energy spacing *ΔE* extracted from **a** as a function of pump spot separation *L*. Error bars are standard deviation of average energy spacing. Red line: the calculated values from theory.

To investigate the dependence of the energy spacing of the quantised state to the trapping potential, we measured the relative energies *δE* (= mode energy − polariton ground state energy) of the quantised states for different pump spot separations *L* (Fig. 2). Although the measurements for different separations were performed at slightly different sample locations, the polariton ground states are all in the range from 2.817 to 2.821 eV. An increasing number of energy levels can be resolved with increasing separation between the two pump spots. For the separation of 8.8 μm, it also follows this trend at lower pump power (Fig. S4). Corresponding results extracted from simulations (solid lines) are in good agreement with the experimental values (solid circles). The average energy spacing for different pump separations (Fig. 2b) shows an inverse dependence of energy spacing on separation, which suggests a stiffer trapping potential as the pump spots get closer. Furthermore, for a given pump separation, we observe that the energy spacing only increases slightly with increasing pump fluence (Supplementary Fig. S4).

### Power dependence of polariton population

**Fig. 3:**
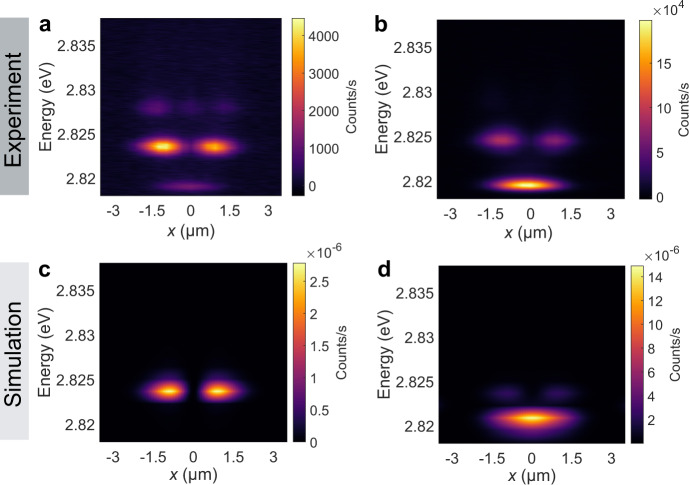
Power dependence of polariton occupation on different quantised states. **a**, **b** Spatially resolved spectra of the cavity excited by two pump spots separated by 5.6 μm at pump powers of 4.7 μW (**a**), 7.4 μW (**b**). **c**, **d** Theoretically calculated spatially resolved spectra considering purely the linear dynamics with *P*_0_ = 12*γ* (**c**), and the occurrence of stimulated energy relaxation with *P*_0_ = 20*γ* and *η* = 10 μm/(meV fs) (**d**).

The dependence of the relative quantum state occupations on pump power while fixing the pump separation at 5.6 μm shows that the quantised states are populated differently at different pump powers (Fig. 3a, b). At 4.7 μW, which is just above the threshold pump power, polariton emission from the *n* = 1 quantum state is dominant, while polaritons tend to occupy the *n* = 0 state at a higher pump power of 7.4 μW. Our theoretical explanation of the different mode occupations employs the GPE by adding the incoherent terms for the driven-dissipative dynamics of the polariton system (Supplementary Section 4). We consider the incoherent driving of an adiabatically eliminated exciton reservoir^33^. The simulations of the driven-dissipative GPE (Eq. (S2)) demonstrate an excellent match with the experiment regarding both mode energies and their occupations (Fig. 3c, d). To determine the origin of power-dependent polariton populations, we theoretically examined different mechanisms, including the blueshift of polariton modes (offset of *V*), optical nonlinearity (nonzero *g*), and stimulated energy relaxation (e.g. phonon scattering)^34–36^. We find that the simulation can reproduce the shift in the mode occupations with increasing pump power only when the effect of stimulated energy relaxation is considered (see Supplementary Information S4). This is different from what has been reported in GaAs cavities, where the self-organised nature of nonlinear polaritons^17^ or the overlap of the mode with the pump spot plays the main role in determining the dominant mode^37^.

### Delocalisation and polariton condensate characteristics in 2D trapping

**Fig. 4:**
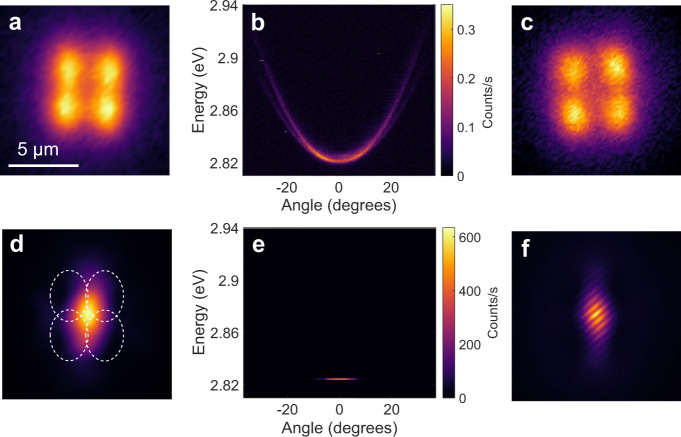
Characterisation of single-mode condensate in two-dimensional trapping. **a–c** Real-space image, angle-resolved PL, and interferogram of the cavity pumped by four laser spots (5.8 μm × 4.4 μm each) with centres located at vertices of a square with edges of 2.8 μm at 0.02*P*_th_. **d–f** Real-space image, angle-resolved PL, and interferogram at 2.1*P*_th_. White dashed ellipses in **d** indicate the positions of pump spots.

In a 1D potential well, although polaritons are well-confined along the direction between two pump spots, they can still leak out in the perpendicular direction (Fig. 1c). The flexibility of all-optical pump control easily allows for 2D confinement of polaritons by shaping the original non-resonant pump into a square array of four pump spots. At low pump power, only incoherent emission at the pump positions is viewed in real space (Fig. 4a). In *k*-space, emission from the lower polariton branch is observed below the threshold (Fig. 4b), with a polarisation splitting at high angles which may be caused by the different penetration depths in the DBRs for different polarisations^38^. Figure 4c shows the interferogram at low pump power measured by a Michelson interferometer with one arm replaced by a retroreflector. No fringes are present as anticipated below the threshold. On increasing the pump intensity, the density of excitons increases, which effectively raises the 2D trap barrier. Polaritons are injected and accumulate at the bottom of the trap until they reach the condensation threshold. Above the threshold, the macroscopic polariton population is fully confined inside the 2D trap created by the four pump spots and only one intense central spatial mode exists (Fig. 4d). It is interesting to note that the confined polariton condensate is delocalised from the excitation profile (white dashed ellipses) by a macroscopic distance, prior to any build-up of coherence in the pump region. The corresponding angle-resolved emission in Fig. 4e reveals the dispersion of a confined polariton condensate, which is 3 meV blueshifted compared with the untrapped polaritons. Figure 4e also confirms that the polariton condensate favours the lowest energy state when the pump separation is close (2.8 μm in both horizontal and vertical directions). Another hallmark of trapped polariton condensates is clearly shown in Fig. 4f, with interference fringes spread over the condensate in the interferogram, indicating a build-up of long-range spatial coherence.

**Fig. 5:**
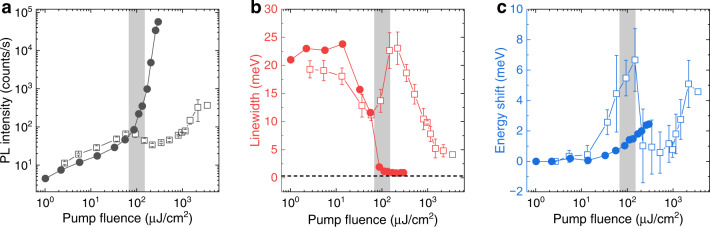
Power dependence of single and square-shaped pump spots. **a–c** PL intensity, linewidth, and peak energy shift of spatially resolved spectra versus pump fluence pumped by single (open squares) and four (solid circles) laser spots. Error bars correspond to the standard deviation over five different sample locations. The grey areas mark the transition to an outwards propagating regime of polaritons when pumped by a single Gaussian spot. The dashed black line in **b** is tangent to the linewidth trend above the threshold pumped by the square array.

To compare the threshold and linewidth of the trapped condensate with an untrapped condensate, we pumped the sample by a single non-resonant Gaussian beam as control. In the control experiment, the PFO cavity was excited by a single Gaussian beam with a spot size of 5.0 μm × 3.9 μm which is similar in area to the individual spots of the square array of four spots. Figure 5a shows the photoluminescence (PL) intensity of spatially resolved spectra for different pump fluences of the two pump patterns. The threshold for polariton condensate excited by four pump spots is found to be 87.6 μJ cm^−2^, corresponding to an excitation density of 7.9 × 10^18^ cm^−3^, beyond which the PL intensity increases nonlinearly. In the case of single Gaussian spot excitation, the PL intensity increases sub-linearly at low pump fluence and starts to drop from 93.8 μJ cm^−2^, then a threshold-like behaviour occurs at 1.2 mJ cm^−2^. The detailed real space images and corresponding spatially resolved spectra at different pump densities are shown in Fig. S6. For this very small Gaussian pump, the above threshold emission becomes distorted into a doughnut-shaped condensate. This could be attributed to the outwards flow of polaritons due to the repulsive potential induced by the pump, which has been seen previously in both inorganic^2^ and organic cavities^39^. The grey region in Fig. 5 marks the transition process to an outwards propagating regime of polaritons, which is irreversible due to permanent bleaching of the sample at pump intensities beyond 10^3^ μJ cm^−2^. Remarkably, the condensation threshold for trapped polaritons is one order of magnitude lower than that for the single Gaussian spot excitation. This can be attributed to the efficient trapping of polaritons when applying the 2D confinement, leading to the required polariton density for the onset of the stimulated scattering process being achieved at lower pump fluence^20^, whereas for the single pump spot, diffusion and ballistic propagation of polaritons away from the pump region hinder the formation of the condensate by depleting the coherent state^40^. We note that the reduction of threshold in our sample is much more dramatic than that in the inorganic samples (~2x)^20^. This could be due to the reduced overlap between the wavefunction of the condensate and the non-resonant exciton reservoir in the trapped geometry which increases the efficiency of polaritons accumulating at the coherent state due to the reduction of bimolecular annihilation^41,42^. The dependence of the polariton condensate threshold on the size of the single Gaussian pump spot was also studied in the same cavity (Figs. S7 and S8). Figure S8 shows that the condensate threshold is much lower when exciting the cavity by a beam with intermediate size (9.3 μm × 8.3 μm) than that for the 2D trapped condensate, the small Gaussian excitation (5.0 μm × 3.9 μm), and the large Gaussian excitation reported previously (diameter of 175 μm)^30^.

Figure 5b shows a clear linewidth narrowing of PL spectra when pumped by the square array (in contrast with the linewidth from the single pump spot excitation that exhibits a sudden broadening when transitioning to an outwards propagating regime of polaritons at 93.8 μJ cm^−2^ and then drops when the condensate forms at the side of the single pump spot). Due to a strong reduction of the decoherence process, caused by the interaction between reservoir and condensate in the case of the trapped condensate, the spectral linewidth above the threshold is only one-fifth of that for the small Gaussian excitation, implying a fivefold longer coherence time^43^. On increasing the pump density, the spectral linewidth remains narrow, confirming that the interaction between the exciton reservoir and condensate has a negligible effect on the phase coherence of the condensate. In contrast, nonlinear effects are evident in the intermediate-size Gaussian excitation condensate, as seen in the spectral broadening when pumping the cavity far above the threshold (Fig. S8b). In the trapped condensate, the energy increases almost linearly from 0.4*P*_th_ to 3.4*P*_th_ with a blueshift of 2.4 meV (Fig. 5c), which is attributed to the reduction of the oscillator strength (‘bleaching’)^32^. For the small Gaussian excitation, the blueshift of peak energy is far steeper in the linear regime as the bleaching is stronger because of the overlap of the emission and excitation spots. The emission peak shifts back when transitioning to an outwards propagating regime of polaritons, giving a net blueshift of 5 meV for the condensate.

### Excited states of polariton condensate in 2D trapping

**Fig. 6:**
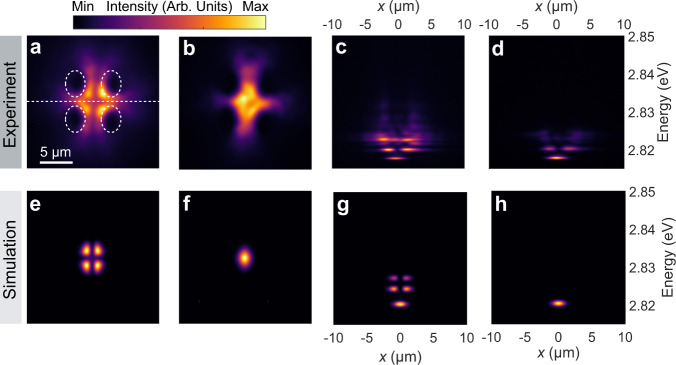
Characterisation of multi-mode condensates in a two-dimensional trap. **a**, **b** Experimental and **e**, **f**, theoretical real-space images of the cavity pumped by four laser spots with centres located at vertices of a square with the edge of 5 μm at 4.1 μW (**a**, **e**), and 6.8 μW (**b**, **f**). White dashed ellipses indicate the positions of pump spots. **c**, **d**, **g**, **h** Corresponding experimental and calculated real-space spectra at 4.1 μW (**c**, **g**) and 6.8 μW (**d**, **h**) along the centre of real-space images indicated by the dashed line in (**a**).

Moreover, we investigated the effect of the 2D trap size on the resulting condensate state. A square array of non-resonant pulsed laser spots with the same individual sizes but 5 μm separation in both horizontal and vertical directions was utilised. Initially, at low pump power, incoherent polariton emission overlapping with the pump positions is clearly observed in Fig. S9a. Figure S9b resolves the centre of the real space image in Fig. S9a, showing relatively broad emission spectra at the pump positions. By raising the pump power, the depth of the optically induced 2D potential trap gradually increases due to the effective increase of exciton density in the reservoir, and polaritons accumulate in the potential trap. Above the threshold, polariton condensates form with four symmetric spatial modes observed in real space (Fig. 6a). On further increasing the pump power and hence increasing the trapping barrier height, the polariton condensates are more firmly confined and drift closer together to form one spatial mode (Fig. 6b). Figure 6c, d show the corresponding spatially resolved spectra in which quantised energy states are clearly displayed. It is noticeable that the state at *n* = 1 is dominant at lower pump power, while higher pump power favours relaxation to *n* = 0, similar to what we observed in 1D traps (Fig. 3). Our theoretical study of excited states in the 2D trap (Fig. 6e–h) employs the same GPE as that in the 1D trap case, showing a relatively good agreement with experimental results. A spatial coherence measurement in Fig. S10 reveals that the individual condensate modes are mutually coherent with each other, indicating phase-locking of the multiple condensate states.

## Discussion

We demonstrate room-temperature organic polariton condensates in 1D and 2D optical potentials created by optically shaping a non-resonant pump laser. In 1D potentials, we observe quantised states in organic microcavities with energy spacings inversely depending on separation. The square-shaped 2D confinement allows for the formation of a polariton condensate spatially separated from the pump region by a macroscopic distance. In our case, this distance is 2–3 μm, showing that polaritons can travel much further than excitons in organic semiconductors. This separation significantly suppresses the decoherence process associated with the exciton reservoir, as evidenced by an order of magnitude lower threshold and higher temporal coherence, as compared to the control Gaussian pump profile. The delocalised polariton condensate in organic microcavities has significance in advancing electrically driven polaritonic devices, as it separates the condensate region from the exciton formation region and so may be harnessed to suppress quenching near metal electrodes. Additionally, by increasing the separation of the 2D trap, polariton condensates in higher-order modes are observed. Selectivity of the condensate state by pump density is observed in both 1D and 2D trapped systems. Theoretical simulations indicate that stimulated energy relaxation leads to lower energy condensate modes at higher pump power, a property that is consistent with the effect of phonon scattering. Our results demonstrate the capability of tailoring and manipulating room-temperature organic polariton condensates by simply configuring the pump geometry, which can be extended to polariton condensate lattices for future quantum applications.

## Methods

### Fabrication

The PFO cavity studied is a *λ*/2 microcavity, which consists of DBRs with 10.5/6.5 pairs on the bottom/top. The individual pairs formed from Ta_2_O_5_/SiO_2_ layers of thicknesses of 46.5/69.7 nm, respectively were deposited by magnetron sputtering. The estimated cavity *Q* factor from reflectivity measurements of the DBR mirrors is 244, corresponding to a cavity photon lifetime of 69 fs. A 140-nm-thick PFO layer was placed at the antinode of the cavity mode with a 10-nm-thick SiO_2_ layer on each side, resulting in a Rabi splitting of 520 meV and cavity detuning of −267 meV. To prepare the PFO layer, the solute (ADS129BE, American Dye Source) was dissolved in toluene at a concentration of 16 mg/mL and stirred overnight at 60 °C. The solution was then heated at 110 °C for 3 min immediately prior to spin-coating to obtain amorphous films.

### Characterisation

The cavity was excited non-resonantly at 343 nm by a vertically polarised pulsed laser (Light Conversion Pharos) with 200 fs pulse width and 5 kHz repetition rate. The original laser beam has a Gaussian profile with a diameter of 4 mm. We note that all beam sizes reported in this work are to be understood as 1/e^2^ width unless specified otherwise. A digital light projector was used to generate patterned laser spots focused on the substrate of the cavity with an aspheric lens (*f* = 4.00 mm, NA = 0.60) near normal incidence. The angle-resolved emission from the cavity was collected by a Fourier imaging microscope with an objective (×40, NA = 0.60). The signal was detected by a charge-coupled device (CCD) spectrometer (Andor Shamrock SR500i) which resolved the emission spectra using an 1800 grooves/mm grating in both energy and angle. The entrance slit was set to 100-μm width. Spatially resolved spectra were recorded by removing one of the Fourier transform lenses. Real-space images were measured by completely opening the entrance slit and aligning the grating to its zeroth order. A Michelson interferometer with one arm replaced by a retroreflector was employed to map the spatial coherence of the emission from the cavity. The pump laser was excluded from all images by a 400-nm long pass filter. All measurements of the cavity were performed at room temperature in air.

### Theoretical calculation

The modes from Fig. 2 were calculated by diagonalisation of $\hat{H}$ on a 1D grid with periodic boundary conditions. The Gaussian pump spots were assumed to have a height of 20 meV and a horizontal standard deviation (1/4 of the 1/e^2^ width) of 1 μm for the lowest two separations, and 1.125 μm for higher separations, with masses extracted from the experimental free polariton dispersion.

For Figs. 1 and 6, we diagonalised on a 2D grid with Dirichlet boundary conditions instead (these ensure confinement of the mode in the transverse direction—an effect that is in practice resulting from the incoherent dynamics that was not considered for these simulations).

The figures of the dynamical simulations as appearing in Fig. 3 average over *N* = 100 realisations in 1D, with the initial state as a vacuum with noise corresponding to an average ${\left|\psi \right|}^{2}=0.1/{{{{{\rm{\mu }}}}}}{{{{{\rm{m}}}}}}$. Since the lifetime of an exciton reservoir is known to exceed that of the cavity polaritons by orders of magnitude^44^, we assume that the steady-state dominates the time-averaged results. In each simulation, we therefore briefly evolve until this steady state has been reached, and then average over a further evolution of 3 ps (fitted to the linewidth of the experiment). The polariton decay rate is *γ*^−1^ = 96 fs, obtained by the cavity loss rate weighed by Hopfield coefficients. We have observed a threshold around ${P}_{0}=10\gamma$. For the figures corresponding to the linear results, we used ${P}_{0}=12\gamma$, while for the result at high driving, we set ${P}_{0}=20\gamma$. The gain saturation ${\sigma }_{0}=47{{{{{\rm{\mu }}}}}}{{{{{\rm{m}}}}}}\cdot \gamma$ was taken in the simulations shown, although similar results are obtained for other values. The cutoff frequency was, as fitted to the experiment (highest modes that are significantly occupied, as seen from Fig. 2), taken to be *Ω* = 15 meV. To study the effect of the drive dependence, with the first hypothesis we used a global blueshift of 1 meV; with the second hypothesis, an optical nonlinearity of *g* = 100 meV μm, and for the third, concluding one *η* = 10 μm/(meV fs). In practice, Eq. (S4) was implemented as ${\left.\frac{{\rm {d}}\psi }{{{\rm {d}}t}}\right|}_{{{\rm {relax}}}}=\frac{+\eta {\hslash }^{2}}{4m}\left({\left|\psi \right|}^{2}{\nabla }^{2}\psi -{\psi }^{2}{\nabla }^{2}{\psi }^{*}\right)$, as obtained from a straightforward calculation that demands *μ* to conserve particle number.

## Supplementary information

Supplementary Information

## Data Availability

The research data generated in this study have been deposited in the University of St Andrews Research Portal [10.17630/46b1551a-f39c-47cf-b16d-44d84350bea4]^45^.

## Code availability

The codes that support the plots within this paper are available from the corresponding author upon reasonable request.
