# Supplementary material for: Optically trapped room temperature polariton condensate in an organic semiconductor
Source: Nat Commun. 2022 Nov 23;13:7191. doi: 10.1038/s41467-022-34440-0 (PMC9691723; doi:10.1038/s41467-022-34440-0)
Supplement: Supplementary file 1 — Supplementary Information [file 41467_2022_34440_MOESM1_ESM.pdf]

Optically trapped room temperature polariton condensate in an organic semiconductor

Mengjie Wei, Wouter Verstraelen, Konstantinos Orfanakis, Arvydas Ruseckas, Timothy C.H. Liew, Ifor D.W. Samuel, Graham A. Turnbull, Hamid Ohadi

1. Linear regime properties

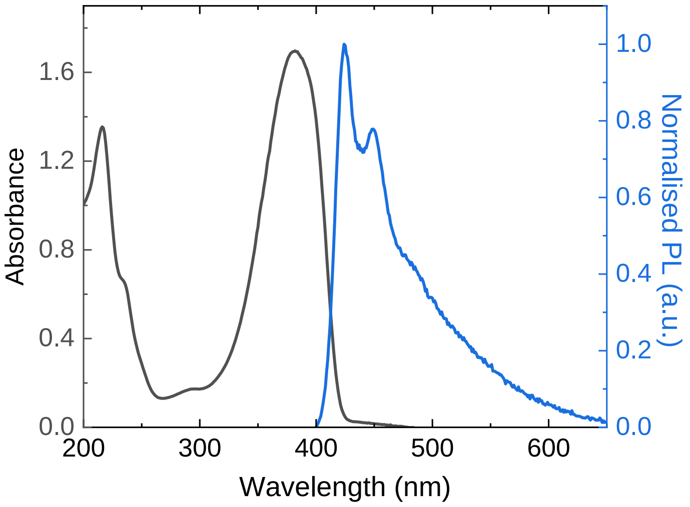

Figure S1. Bare film properties. Absorption and emission spectra of 135-nm-thick glassy-phase PFO film on fused silica substrate.

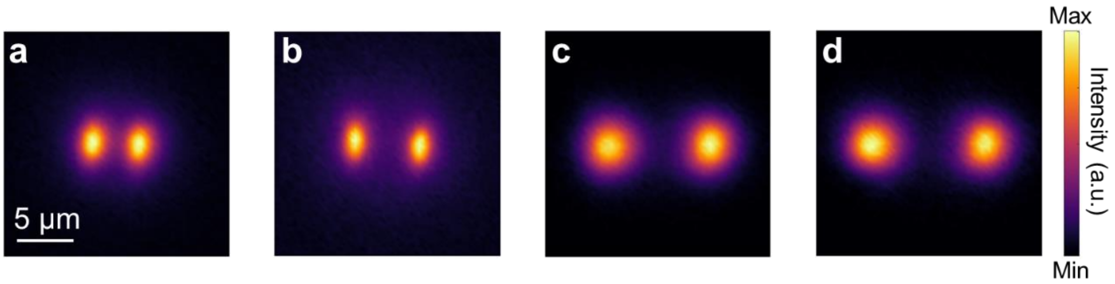

Figure S2. Real space images of one-dimensional trapping below threshold. a-d, Real-space images of the cavity excited by two pump spots 4.1 μm (a), 5.6 μm (b), 8.8 μm (c), 10 μm (d) at very low pump intensity.

2. Characterisation of 1D trapping for more pump separations

Figure S3a-c shows real-space images of the cavity above threshold excited by two pump spots separated by 5 µm, 6.6 µm, and 7.2 µm. By resolving the line between the centres of two pump spots, the energy distribution of polaritons in the 1D potential trap is shown in Fig. S2d-e. By controlling the separation between the pump spots, the shape of the 1D potential is directly modified, leading to different numbers of quantum states trapped inside the potential. Apparently, for shorter separations, fewer condensate states can be observed with polaritons occupying lower energy states, whereas for longer separations, more quantised states are resolved, and polaritons favour the higher energy states at just above threshold.

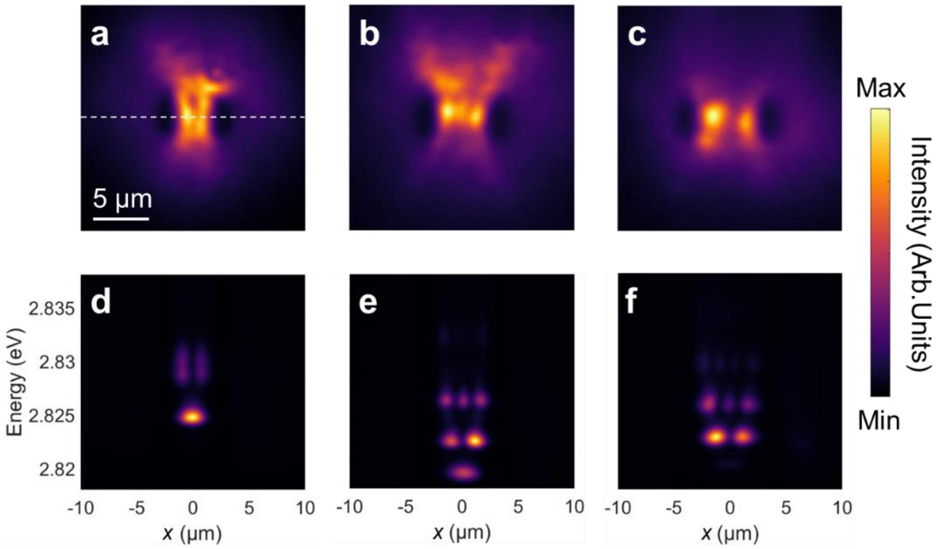

Figure S3. Characterisation of one-dimensional trapping with more different separations. a-c, Real-space images of condensate emission for two 6.6 µm × 4.5 µm pump spots separated by 5 µm (a), 6.6 µm (b), and 7.2 µm (c). **d-f,** Corresponding real-space spectra along the centre of real-space images indicated by dashed line in (a).

3. Power dependence of quantised states in 1D trap

While the energy spacing of quantised states shows an inverse dependence on the pump separation, increasing the pump power of the two pump spots while keeping the separation constant only slightly increases the energy spacing (Fig. S4).

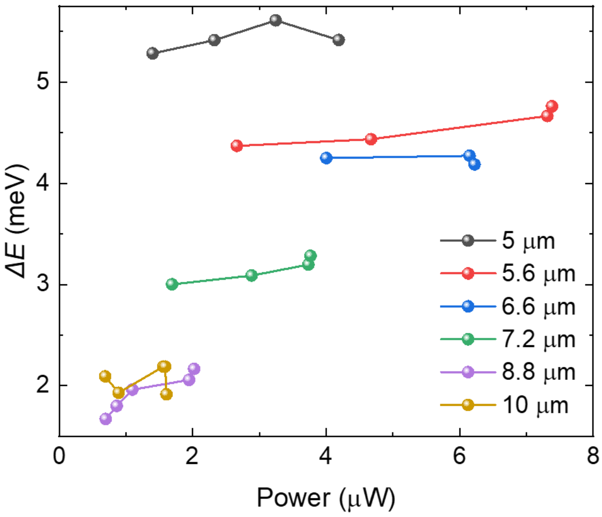

Figure S4. Power dependence of energy spacing. Energy spacing of quantised states for different pump separations at different pump powers above threshold.

4. Theoretical calculations for power dependence of polariton populations

We assume driving of the polariton condensate to have the same spatial profile $P(\mathbf{x})$ as the pump spots, disregarding the small diffusion of initially excited hot excitons. The reservoir contains many modes at different frequencies below the external drive frequency, and through intractable dynamics, excitons will redistribute among those and partially thermalise.[1] Ultimately, this leads to a large occupation of a low-energy polariton mode, where condensation will take place. For the case of multimode polariton condensation as studied in this work, it can be expected that lower energy modes experience a higher driving in the GPE framework. Assuming driving strength that decreases linearly with frequency up to a cut-off Ω, this gives a driving contribution[2]

$$P(\mathbf{x})\left(1-\frac{i\hbar}{\Omega}\frac{d\psi(\mathbf{x},t)}{dt}\bigg|_{coh}\right) \tag{S1}$$

In addition, we consider uniform incoherent losses through the mirrors at rate γ. Finally, we note that steady-state occupations above threshold are reached because of saturation of the exciton reservoir. Its effect can be included in the GPE by an incoherent nonlinearity $\sigma(\mathbf{x})$ (''nonlinear loss'') with the same spatial dependence as $P(\mathbf{x})$, and height σ_0.

Putting everything together, we obtain the driven-dissipative GPE

$$\frac{d\psi(\mathbf{x},t)}{dt}=\left(\frac{-i}{\hbar}-\frac{P(\mathbf{x})}{2\Omega}\right)\widehat{H}_{\psi,\mathbf{x}}\psi(\mathbf{x},t)+\frac{P(\mathbf{x})-\gamma-2\sigma(\mathbf{x})|\psi(\mathbf{x},t)|^2}{2}|\psi(\mathbf{x},t)|^2 \tag{S2}$$

The frequency-dependent driving equation (S1), naively, seems to suggest relatively higher driving of lower energy modes and thus a mode occupation that decreases monotonically with these energies. However, it is also important to consider a complementary mechanism: the overlap of the mode with the pump spot

$$\int d\mathbf{x}|\psi_n(\mathbf{x})|^2 P(\mathbf{x}) \tag{S3}$$

which will in general be higher for modes of higher n. Indeed, Askitopoulos *et al.* have reported that in a circular trap in a GaAs microcavity, this effect alone has led to a maximal occupation that always takes place at the mode of highest n that fits in the trap.[3] The relative intensities of the different modes are thus determined by an interplay of these two mechanisms. Although the frequency-dependent driving equation (S1) is known to be present already in inorganic polaritons,[1,2] we might expect it to be more pronounced in organic polariton experiments that operate at room temperature[4] and as such more susceptible to fluctuations from the environment, hence the different end result with respect to that reported in GaAs microcavity.[3]

To perform these dynamical simulations, we also note that equation (S2) disregards spontaneous emissions in the condensate and the fluctuations they induce. This is typically justified for large occupations when stimulated processes dominate. However, for the initial evolution from an empty cavity, this omission is troublesome, as stimulated processes are absent. One option would be to add noise to equation (S2) explicitly in some form,[5,6] however, the resulting stochastic differential equations are numerically more demanding.[7] Instead, we opt to add a small amount of noise to the initial vacuum state to break the symmetry.

The increase of the pump power leads to a higher occupation of the lower n mode as shown in Fig. 3b, which cannot be explained by equation (S2) alone, as the gain saturation σ cannot be expected to lead to non-monotonic behaviour. Unlike in inorganic microcavities, the potential V remains almost constant with P_0 above threshold in the organic case.[8] We will consider three different hypotheses for the observed behaviour.

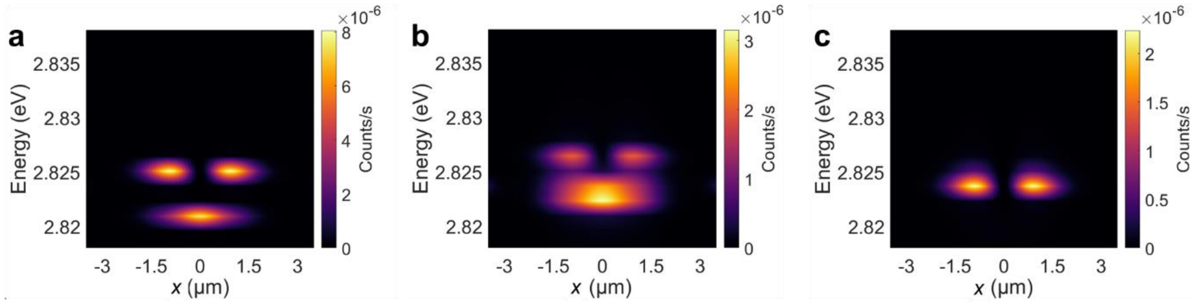

Figure S5. Simulation of relative polariton population in different situations. a-c, Spatially resolved spectra of the cavity excited by two pump spots separated by 5.6 μm at the condition of $P_0 = 20\gamma$ with a global blueshift of 3 meV (a), $P_0 = 20\gamma$ and $g = 100\ \mathrm{meV}\cdot\mu\mathrm{m}$ (b), and $P_0 = 12\gamma$ and $\eta = 10\ \mu\mathrm{m}/(\mathrm{meV}\cdot\mathrm{fs})$ (c).

First, we notice that the increasing drive does result in an overall blueshift of the modes that is experimentally observable in Fig. 3a and 3b. Denoting the height of the Gaussian pump profile by P_0, we might then envision a shift of the frequencies $\Delta(P_0)$ to the bare mode frequencies ω_n such that $\omega_n + \Delta\left(P_0^{(1)}\right) < \Omega$ while $\omega_n + \Delta\left(P_0^{(2)}\right) > \Omega$, for two different pump powers $P_0^{(1)}$, $P_0^{(2)}$, i.e., the blueshift brings the modes to frequencies that are no longer driven. However, it is clear from Fig. 3a and 3b that the experimentally observed blueshifts are definitely too low to account for the phenomenon. Assuming a global blueshift of 1 meV, which is certainly more than observed, and $P_0 = 20\gamma$, where γ is the polariton decay rate, this fails to reproduce Fig. 3b as shown in Fig. S5a.

Second, although true optical nonlinearity is known to be negligible ($g = 0$ as discussed above) for organic polaritons, effective mechanisms, such as quenching of the Rabi splitting, may mimic its effect[8] and result in the appearance of a finite contribution $g = g_{eff}$ (which would be in addition to quenching of Rabi splitting contributing to $V(\mathbf{x})$). However, as observed in Fig. S5b with $P_0 = 20\gamma$ and a large optical nonlinearity of $g = 100\ \mathrm{meV}\cdot\mu\mathrm{m}$ added, this situation does not occur; instead the finite nonlinearity merely leads to an energy shift as discussed above and blurring of the energy levels.

Third, we consider the occurence of stimulated energy relaxation at rate η, such as appearing from phonon scattering.[9–11] The significance of a stimulated mechanism would increase with P_0. To take this effect into account, a term

$$\left.\frac{d\psi(\mathbf{x},t)}{dt}\right|_{relax} = -\eta|\psi(\mathbf{x},t)|^2\left(\widehat{H}_{\psi,\mathbf{x}} - \mu(\mathbf{x},t)\right)\psi(\mathbf{x},t) \quad \text{(S4)}$$

is added to the right-hand side of equation (S2), where $\mu(\mathbf{x},t)$ is an effective chemical potential that ensures that the number of particles is conserved when they relax to lower modes. Figure 3d shows that this properly reproduces the shift in mode occupations with increasing pump power towards lower n, as seen in the experiment.

Figure S5c shows the calculation with $P_0 = 12\gamma$ and $\eta = 10\ \mu\text{m}/(\text{meV}\cdot\text{fs})$. We observe that because of the lower driving, it retrieves the linear result matching Fig. 3a, even if η is fixed.

5. Single spot condensation

Figure S6 shows the characterisation of the cavity excited by a small Gaussian laser beam (5.0 µm × 3.9 µm). At low pump fluence, incoherent emission is observed in both space (Fig. S6a) and energy (Fig. S6d). On increasing the pump fluence, photodegradation at the pump location is clearly seen as a hole in real space image (Fig. S6b) with no photoluminescence (PL) resolved at the corresponding position in Fig. 5e. Due to strong repulsive interactions between polaritons at high polariton density, the polaritons expand outwards.[12–14] Finally, a polariton condensate forms at the side of the pump spot (Fig. S6c) with spectrum narrowing observed in Fig. S6f.

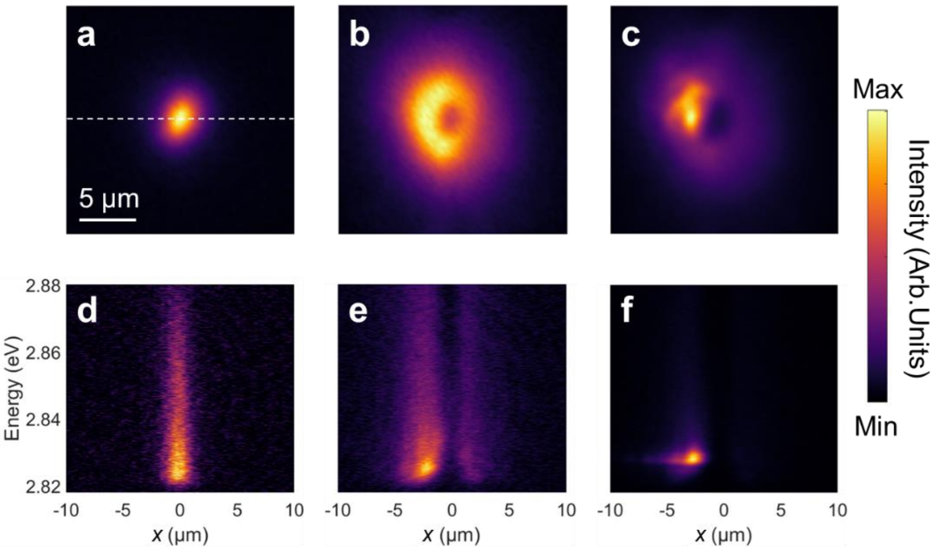

Figure S6. Characterisation of the cavity excited by a small Gaussian laser beam. a-c, Real-space images of the cavity pumped by a Gaussian beam (5.0 µm × 3.9 µm) at 2.7 µJ cm-

[2] (a), 828 μJ cm^{-2} (b), 2.2 mJ cm^{-2} (c). **d-f,** Corresponding real-space spectra along the centre of real-space images indicated by dashed line in (a).

Interestingly, when exciting the cavity by an intermediate-sized Gaussian pump beam (9.3 μm × 8.3 μm) with size similar to the sum of the square-shaped four pump spots, a localised polariton condensate is formed as reported in previous work, although with less disorder and instability (Fig. S7).[4,15,16]

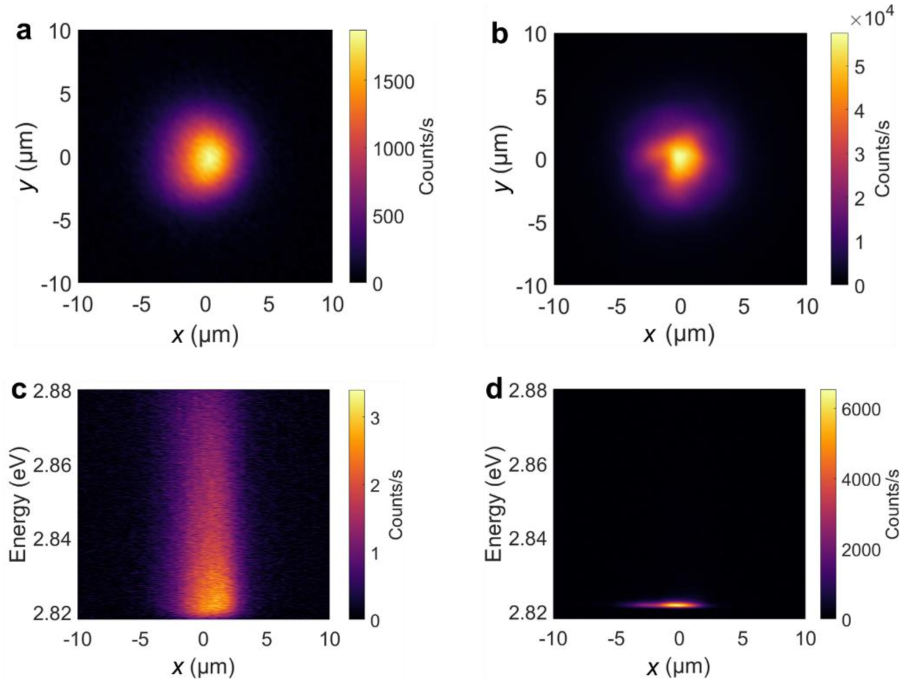

Figure S7. Characterisation of the cavity excited by an intermediate Gaussian laser beam. a, b, Real-space images of the cavity pumped by a Gaussian beam (9.3 μm × 8.3 μm) at 0.48 μJ cm^{-2} and 34.7 μJ cm^{-2}. **c, d,** Corresponding real-space spectra on the line of $y = 0$ of real-space images.

Figure S8 quantifies the power dependence of the polariton condensate excited by this intermediate-sized Gaussian beam. The threshold of polariton condensate is at 21.4 μJ cm^{-2} where the PL intensity transits from the sublinear to the superlinear regime in Fig. S8a. It is worth noting that the linewidth of PL spectra below threshold is broader than that excited by four pump spots, resulting from the interaction between polaritons and the overlapping exciton reservoir. When pumped above threshold, the linewidth dramatically reduces to 1.28

meV, which is 1.5 times that of the 2D trapped condensate and exhibits a slight broadening due to polariton-polariton and polariton-exciton interactions induced decoherence process.[17,18] The localised condensate shows a blueshift of 2.1 meV at 3 P_{th} (Fig. S8c).

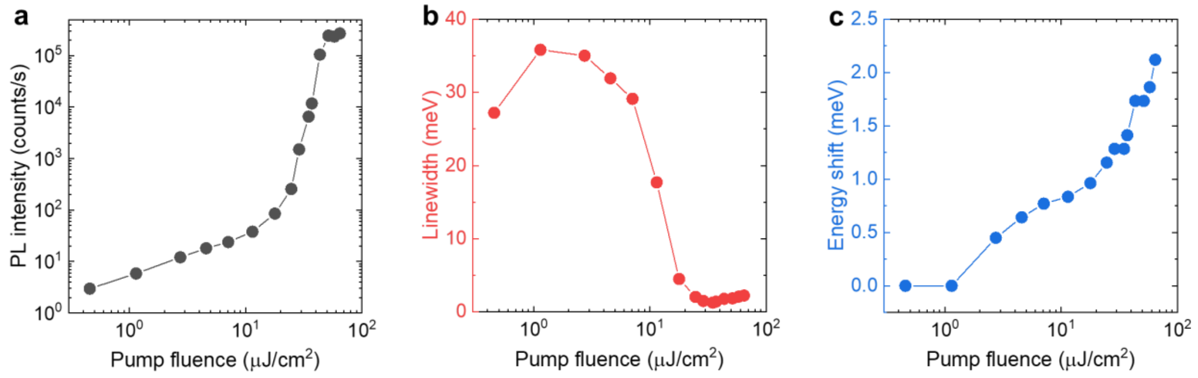

Figure S8. Power dependence of an intermediate Gaussian spot. a-c, PL intensity, linewidth, and peak energy shift of spatially resolved spectra versus pump fluence.

6. Multi-mode condensate in 2D trapping

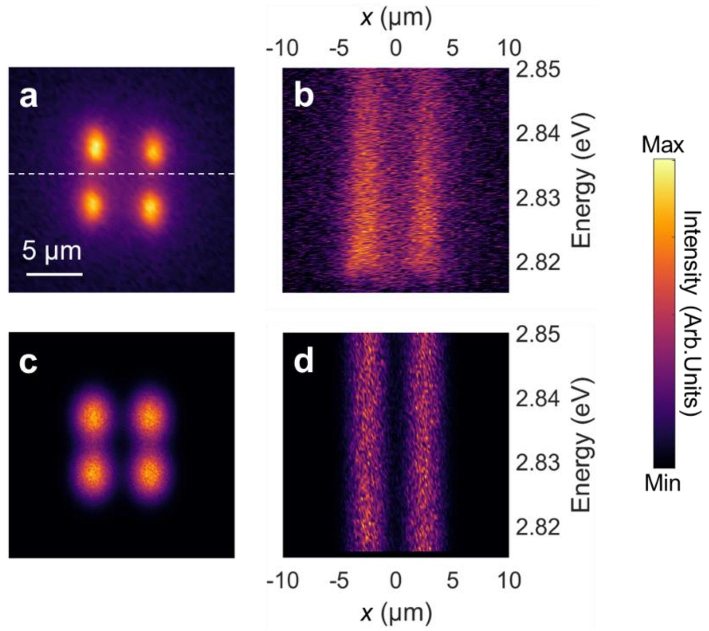

Figure S9. Experimental and theoretical demonstration of polariton emission in two-dimensional trapping below threshold. a,b Experimental real space image (a) and spatially resolved spectrum (b) along the white dashed line in (a) pumped by four laser spots with centers located at vertices of a square with edge of 5 μm at 4.1 nW. **c,d,** Corresponding simulation results.

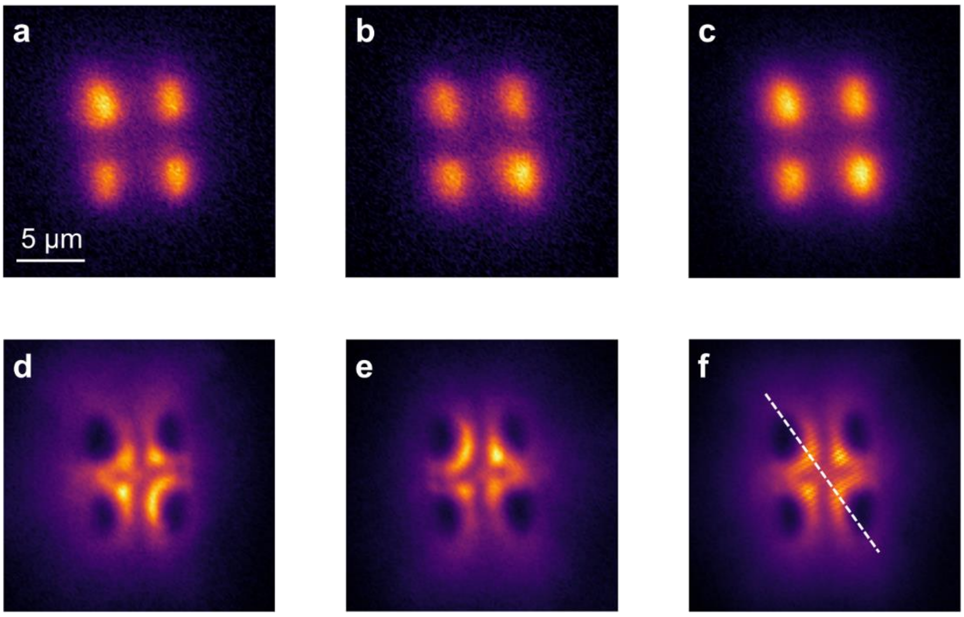

Figure S10. Spatial coherence of multi-mode condensate in two-dimensional trapping. a-c, Real-space images of the cavity pumped by four laser spots with centres located at vertices of a square with edge of 5 μm at 17 nW from the aluminium mirror arm (a), the retroreflector arm (b), and the overlapping image (c). **d-e,** Corresponding images recorded at 2.2 μW.

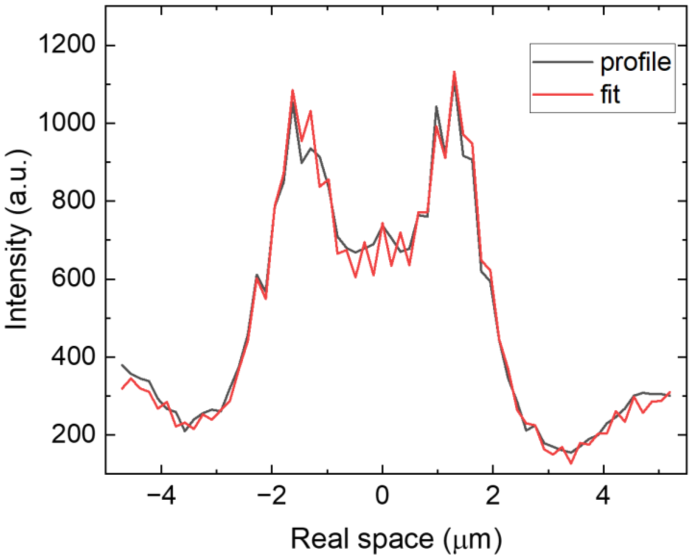

Figure S11. Fringe visibility of spatial coherence of multi-mode condensate in two-dimensional trapping. Black line is the profile of the dashed line in Figure S10f, red line is the fit using Equation A1 in Ref. 18, giving a fringe visibility of around 28%.

References

1. Porras, D., Ciuti, C., Baumberg, J. J. & Tejedor, C. Polariton dynamics and Bose-Einstein condensation in semiconductor microcavities. *Phys. Rev. B* **66**, 853041–8530411 (2002).
2. Wouters, M. & Carusotto, I. Superfluidity and critical velocities in nonequilibrium Bose-Einstein condensates. *Phys. Rev. Lett.* **105**, 9–12 (2010).
3. Askitopoulos, A. et al. Robust platform for engineering pure-quantum-state transitions in polariton condensates. *Phys. Rev. B* **92**, 1–6 (2015).
4. Wei, M. et al. Low-threshold polariton lasing in a highly disordered conjugated polymer. *Optica* **6**, 1124-1127 (2019).
5. Wouters, M. & Savona, V. Stochastic classical field model for polariton condensates. *Phys. Rev. B* **79**, 1–10 (2009).
6. Henry, C. H. Theory of the linewidth of semiconductor lasers. *IEEE J. Quantum Electron.* **18**, 259-264 (1982).
7. Milstein, G. N. & Tretyakov, M. V. Stochastic numerics for mathematical physics. (Springer Science & Business Media, 2013).
8. Yagafarov, T. et al. Mechanisms of blueshifts in organic polariton condensates. *Commun. Phys.* **3**, 1–10 (2020).
9. Wouters, M. Energy relaxation in the mean-field description of polariton condensates. *New J. Phys.* **14**, 1-12 (2012).
10. Wouters, M., Liew, T. C. H. & Savona, V. Energy relaxation in one-dimensional polariton condensates. *Phys. Rev. B* **82**, 1–6 (2010).
11. Antón, C. et al. Energy relaxation of exciton-polariton condensates in quasi-one-dimensional microcavities. *Phys. Rev. B* **88**, 1–13 (2013).
12. Savvidis, P. G. et al. Angle-resonant stimulated polariton amplifier. *Phys. Rev. Lett.* **84**, 1547–1550 (2000).
13. Kavokin, A., Baumberg, J. J., Malpuech, G. & Laussy, F. P. Microcavities. *Oxford University Press* **6**, (2011).
14. Wertz, E. et al. Spontaneous formation and optical manipulation of extended polariton condensates. *Nat. Phys.* **6**, 860–864 (2010).
15. Daskalakis, K. S., Maier, S. A., Murray, R. & Kéna-Cohen, S. Nonlinear interactions in an organic polariton condensate. *Nat. Mater.* **13**, 271–8 (2014).
16. Rajendran, S. K. et al. Low Threshold Polariton Lasing from a Solution-Processed Organic Semiconductor in a Planar Microcavity. *Adv. Opt. Mater.* **1801791**, 1–7 (2019).

17. Porras, D. & Tejedor, C. Linewidth of a polariton laser: Theoretical analysis of self-interaction effects. *Phys. Rev. B* **67**, 1–4 (2003).
18. Kim, S. et al. Coherent polariton laser. *Phys. Rev. X* **6**, 1–9 (2016).
